# Corrigendum: Constraint-induced movement therapy enhances angiogenesis and neurogenesis after cerebral ischemia/reperfusion

**DOI:** 10.4103/NRR.NRR-D-25-01000

**Published:** 2026-02-11

In the article titled “Constraint-induced movement therapy enhances angiogenesis and neurogenesis after cerebral ischemia/reperfusion,” published in *Neural Regeneration Research* (Zhai and Feng, 2019), there are errors in the selection of images for Figure 12A made by the authors during the assembling process.

In Figure 12A displaying representative immunohistochemistry images of VEGFR2-positive cells in the ischemic boundary zone of the impaired cortex at 4 weeks after middle cerebral artery occlusion in Figure 12A, the image labeled “Sham1” is incorrect. Below is the correct Figure 12A:


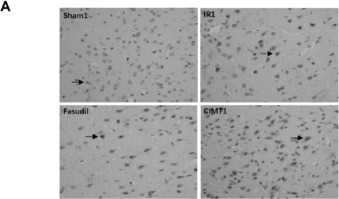


The authors confirm that all results and conclusions of this article remain unchanged. They apologize for any inconvenience this correction may cause for readers and editors of *Neural Regeneration Research*.

The online version of the original article can be found at doi: 10.4103/1673-5374.257528.
